# Clinician-Reported Respiratory Viral Testing and Intended Management of Community-Acquired Respiratory Viral Infections in Haematology and Haematopoietic Cell Transplantation: An International Exploratory Survey

**DOI:** 10.3390/diagnostics16172786

**Published:** 2026-08-30

**Authors:** Justin R. du Toit, Tobias R. Neijzen, Sjoerd van der Bie, Steven F. L. van Lelyveld, Aart Beeker, Karlijn J. van Stralen, Odette M. Vlek, Estelle R. Verburgh, Eric van Gorp, Jurjen Versluis, Marco Goeijenbier

**Affiliations:** 1 Wits Donald Gordon Medical Centre, School of Clinical Medicine, Faculty of Health Sciences, University of the Witwatersrand, Johannesburg 2000, South Africa; 2 Department of Viroscience, Erasmus Medical Center, 3015 GD Rotterdam, The Netherlands; e.vangorp@erasmusmc.nl; 3 Department of Intensive Care, Spaarne Gasthuis Hospital, 2134 TM Hoofddorp, The Netherlands; tneijzen@spaarnegasthuis.nl (T.R.N.); svanderbie@spaarnegasthuis.nl (S.v.d.B.); 4 Department of intensive Care, Erasmus Medical Center, 3015 GD Rotterdam, The Netherlands; 5 Department of Internal Medicine, Spaarne Gasthuis Hospital, 2134 TM Hoofddorp, The Netherlands; s.van.lelyveld@spaarnegasthuis.nl (S.F.L.v.L.); abeeker@spaarnegasthuis.nl (A.B.); 6 Spaarne Gasthuis Academy, Spaarne Gasthuis Hospital, 2134 TM Hoofddorp, The Netherlands; kvanstralen@spaarnegasthuis.nl; 7 Independent Researcher, Ovisionconsulting, Badhoevedorp, 1171 DX Amsterdam, The Netherlands; odette@ovisionconsulting.nl; 8 Department of Medicine, Division of Clinical Haematology, University of Cape Town, Cape Town 7925, South Africa; estelle.verburgh@uct.ac.za; 9 Erasmus MC Cancer Institute, University Medical Center Rotterdam, 3015 GD Rotterdam, The Netherlands; j.versluis.1@erasmusmc.nl

**Keywords:** respiratory syncytial virus, haematology, haematopoietic cell transplantation, ribavirin, community-acquired respiratory viral infections

## Abstract

**Background:** Community-acquired respiratory viruses (CARVs) cause significant morbidity and mortality in patients with haematological malignancies and those undergoing haematopoietic cell transplantation (HCT). Despite this, clinical management remains variable. This study evaluated clinicians’ awareness, diagnostic practices, and treatment approaches to major respiratory viruses in this high-risk population. **Methods:** An international electronic survey using non-probability convenience sampling was conducted among haematology, oncology, intensive care and emergency medicine physicians. Four real-life clinical cases were converted into standardised vignettes covering human metapneumovirus (HMPV), respiratory syncytial virus (RSV), human parainfluenza virus (HPIV) and influenza A virus (IAV). The questionnaire assessed reported testing strategies and intended management, including antiviral and immunoprophylactic interventions. Responses were analysed descriptively; no inferential between-group comparisons were performed. **Results:** Ninety-eight clinicians from 26 countries responded. Diagnostic approaches, including testing indications and panel selection, varied among respondents. For HMPV and HPIV, respondents mainly reported supportive care, immunoglobulin replacement, and infection-control measures in the absence of established pathogen-specific interventions. RSV practice was heterogeneous: systemic ribavirin was considered by 45/96 (46.9%) respondents, including 34/96 (35.4%) when guided by an immunodeficiency score. IAV management was more consistent, with reported availability of vaccination and antiviral therapy and frequent use of post-exposure prophylaxis. **Conclusions:** Within this international convenience sample, clinicians reported variation in respiratory viral testing and in how results informed intended management. Because sampling was non-probability-based and respondent groups were small and unevenly distributed, the findings should not be interpreted as representative estimates of international practice. IAV testing was most consistently linked to established treatment and prevention, RSV showed greater therapeutic and preventive heterogeneity, and HMPV/HPIV testing primarily informed infection control, monitoring and antimicrobial stewardship.

## 1. Introduction

Respiratory viral infections are a major cause of morbidity and mortality in immunocompromised patients, particularly those with haematological malignancies and recipients of haematopoietic cell transplantation (HCT) [1]. Community-acquired respiratory viruses (CARVs), including respiratory syncytial virus (RSV), influenza A virus (IAV), human metapneumovirus (HMPV), human parainfluenza virus (HPIV) and seasonal coronaviruses, may cause severe disease in this population and can progress rapidly from upper to lower respiratory tract infection [1].

Recent data further emphasise the burden of RSV in adult and immunocompromised populations. In a comparative ICU cohort of severe RSV, HMPV, IAV and HPIV infections, haematological malignancies were more common among patients with RSV, and hospital mortality in the RSV group was 25.5% [2]. In a Spanish population-based analysis of 20,695 adults hospitalised with RSV, leukaemia was independently associated with in-hospital mortality (adjusted odds ratio 3.31, 95% confidence interval 2.11–4.59) [3]. A recent systematic review likewise documented substantial proportions of hospitalisation, ICU admission and mechanical ventilation among immunocompromised adults with RSV [4], while comparative data in older hospitalised adults demonstrated major short-term morbidity and mortality from RSV, broadly comparable to influenza [5]. RSV is therefore particularly relevant to haematology and HCT practice [6], and prospective HCT data link post-transplant respiratory viral infections, including RSV, to subsequent pulmonary impairment and mortality [7].

The introduction of multiplex polymerase chain reaction (PCR) platforms and point-of-care testing (POCT) has improved the detection of respiratory viruses in clinical practice and can shorten time to diagnosis [8]. However, availability, turnaround time, testing indications and diagnostic panel selection vary substantially between centres. These differences may influence both the likelihood of identifying a viral pathogen and the way in which results are applied in clinical decision-making.

Importantly, the clinical consequence of a positive result differs by virus. IAV detection usually leads to clear antiviral treatment and post-exposure prophylaxis decisions. RSV may prompt risk stratification and selected use of antiviral or immunoprophylactic strategies, although practice remains heterogeneous and high-quality evidence is limited [9,10,11,12,13]. In contrast, detection of HMPV or HPIV more commonly informs infection-control measures, monitoring and supportive care, given the lack of widely available targeted therapies [13,14].

This study aimed to describe reported respiratory viral testing and intended management among clinicians caring for patients with haematological malignancies, including HCT and non-HCT populations. Using an international case-based questionnaire distributed to haematology, intensive care and emergency medicine physicians, we sought to characterise variation in testing strategies, panel selection and stated responses to diagnostic results in this high-risk population.

## 2. Materials and Methods

The four clinical vignettes were derived from actual patients with haematological malignancies or previous HCT who had PCR-confirmed community-acquired respiratory viral infections. Rather than constructing hypothetical scenarios, these cases were converted into standardised questionnaire vignettes while preserving the central diagnostic and therapeutic dilemmas encountered in clinical practice. All potentially identifying information was removed or modified before inclusion.

The questionnaire was developed in collaboration with O.M.V., who holds an MSc in Communication Science and has 18 years of professional experience in market research, including questionnaire design, survey methodology and interpretation of quantitative survey findings. The draft questionnaire subsequently underwent multidisciplinary expert review. K.S., an epidemiologist with extensive international expertise in study design, reviewed its epidemiological structure and interpretability. Clinical content and plausibility were reviewed by two haematologists with HCT expertise, a board-certified infectious diseases specialist with more than 10 years of specialist experience and an intensivist with 5 years of specialist experience. These reviewers were selected to combine survey methodology, epidemiological, study design and relevant clinical expertise. The review focused on clarity, relevance, interpretability and fidelity to real-world clinical decision-making, and revisions were incorporated iteratively before dissemination.

The questionnaire was designed as an exploratory, case-based assessment of reported clinical practice rather than as an instrument intended to measure a latent construct or psychometric scale. It was therefore not subjected to formal psychometric validation or quantitative content-validity assessment. The final questionnaire is provided in Supplementary File S1.

The questionnaire assessed respiratory viral testing strategies, including indications for testing and diagnostic panel selection and intended responses to positive results. Management questions addressed antiviral and immunoglobulin use, immunisation, infection-control measures and antibiotic management.

Using convenience sampling, potential participants—clinical haematologists, intensive care specialists and emergency medicine physicians, including trainees—were approached between 1 September and 31 October 2025. Potential respondents were identified through professional societies and established clinical networks and were contacted by email and, in some instances, through individual WhatsApp messages or relevant professional WhatsApp groups. WhatsApp was used solely for initial contact; no questionnaire responses were collected through WhatsApp. All formal invitations were subsequently sent by email and contained a link to the electronic questionnaire hosted in ResearchManager. Recruitment targeted approximately 100 clinicians with relevant experience in managing immunocompromised patients with respiratory viral infections. Invitations were distributed until this target was reached; 650 individual email invitations had been sent when recruitment closed. The mailing list was compiled through the European Society for Blood and Marrow Transplantation Trainee Committee and the South African Stem Cell Transplant Society, with additional contacts through international clinical networks. Questionnaire responses were entered directly into ResearchManager and stored in pseudonymised form within the secure platform. Contact details used for recruitment were kept separate from survey responses, and the analytical dataset contained no directly identifying information. Institution identifiers were not collected. Participation was voluntary, and participants were informed before beginning the questionnaire that completion and submission constituted informed consent for participation and use of the survey data for research purposes.

Responses were analysed descriptively and are reported as n/N (%), using the item-specific number of valid responses as the denominator. Missing responses were excluded from item-specific denominators, and percentages for multiple-response questions may exceed 100%. Given the non-probability sampling design and the small and unevenly distributed respondent groups across specialties, geographical regions and country income groups, no inferential between-group comparisons were performed. These characteristics were reported descriptively to characterise the survey sample. Respondent-level transplant-practice status was not collected; therefore, transplant-versus-non-transplant comparisons were not performed.

The study was approved by the Ethics Committee of Spaarne Gasthuis on 7 July 2025 (approval number 2025.0091).

During preparation of this manuscript, the authors used GPT-5.6 Sol and Paperpal Preflight to improve readability and language. The authors subsequently reviewed and edited the content as required and take full responsibility for the final publication.

## 3. Results

### 3.1. Characteristics of Survey Respondents (N = 98)

A total of 98 clinicians completed the questionnaire (98/650; 15.1% response proportion). Clinical haematology specialists or trainees comprised 64/98 (65.3%) respondents. Respondents represented 26 identifiable countries across six world regions; 63/98 (64.3%) were based in Europe and 19/98 (19.4%) in Africa, with smaller representation from South America (6/98, 6.1%), North America (4/98, 4.1%), Asia (3/98, 3.1%), and Oceania (2/98, 2.0%). Country was missing for one respondent (1.0%). Using World Bank fiscal year 2026 classifications [15], 69/98 (70.4%) respondents were from high-income countries, 27/98 (27.6%) from upper-middle-income countries, and 1/98 (1.0%) from a lower-middle-income country; no respondents were from low-income countries, and one country response (1.0%) could not be classified. Median reported clinical experience was 15 years (interquartile range, 8.75–25; 96/98 responses). Participant characteristics are summarised in Table S1, with country-level counts in Table S2 in the Supplementary Materials.

### 3.2. Diagnostic Testing Strategies

**Figure 1:**
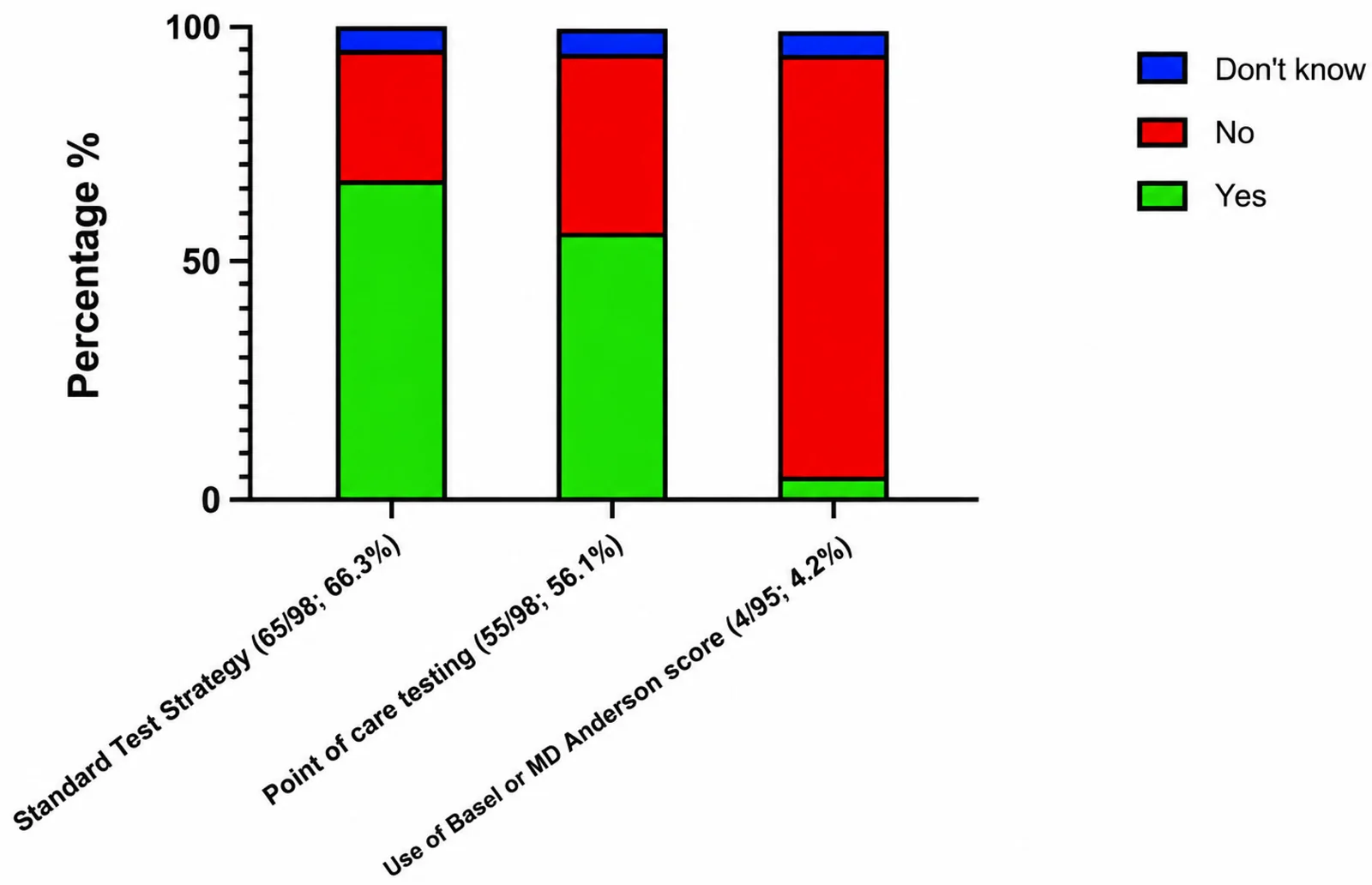
Respiratory viral testing strategies and use of immunodeficiency scoring systems. Bars show reported use of a standardised testing strategy, reported availability of point-of-care testing at respondents’ institutions, and use of the Basel framework or MD Anderson Immunodeficiency Scoring Index (ISI); uncertain and missing responses are shown where applicable.

Standardised testing protocols were reported by 65/98 (66.3%) respondents, while 55/98 (56.1%) reported POCT availability at their institutions; only 4/95 (4.2%) respondents to the risk-score item reported routine use of the Basel framework or MD Anderson Immunodeficiency Scoring Index (ISI) [10,16] (Figure 1).

### 3.3. Case 1—HMPV

A 55-year-old female presented to the emergency department with progressive dyspnoea. Her history included hypertension, acute myeloid leukaemia (AML) treated with allogeneic HCT, and ongoing immunosuppression for graft-versus-host disease.

**Figure 2:**
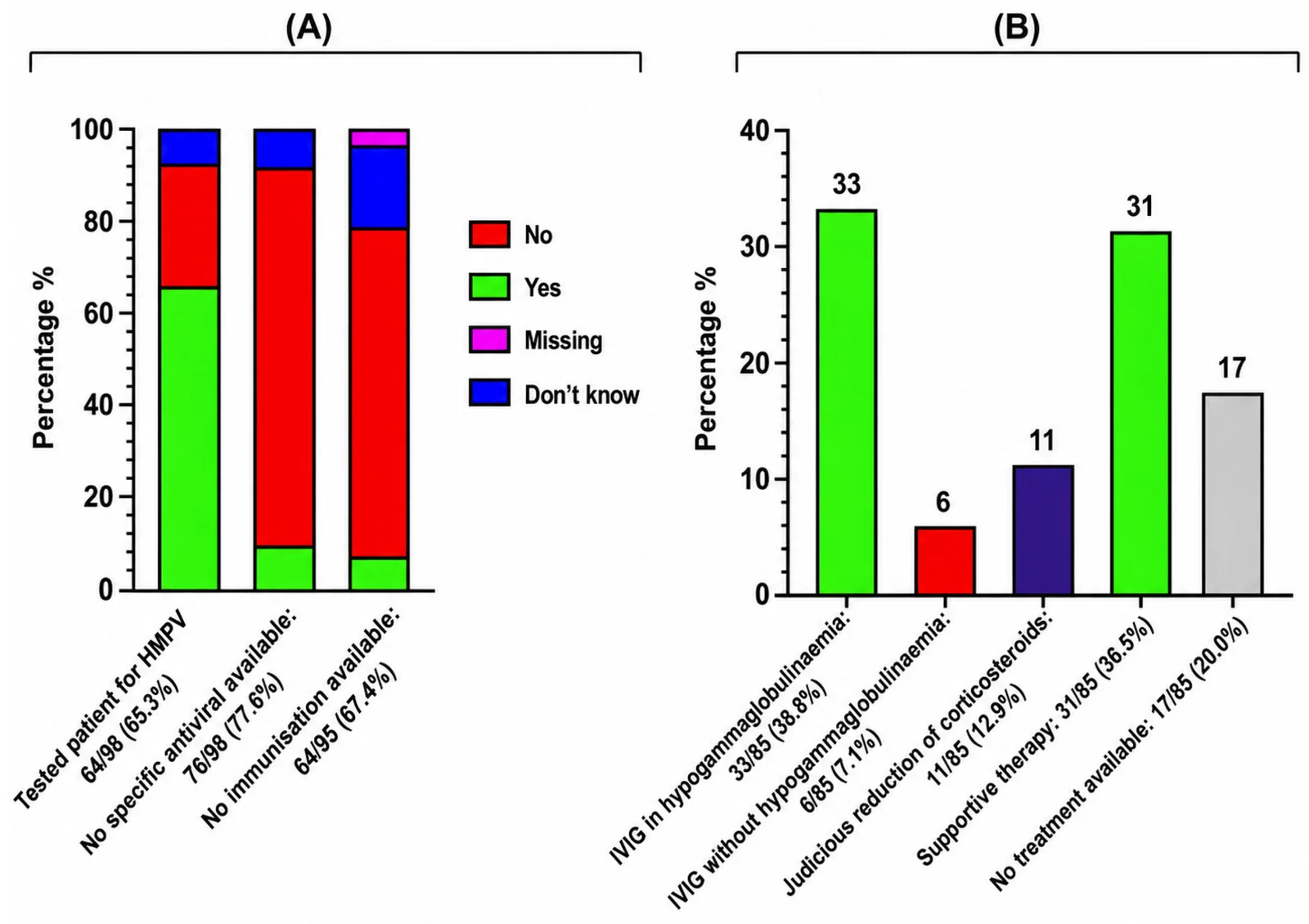
Human metapneumovirus (HMPV) testing, antiviral/immunisation availability, and management responses. (**A**) Testing and reported availability of HMPV-specific antiviral therapy and immunisation. (**B**) Multiple-response management options. Percentages use item-specific denominators.

For the HMPV vignette, 64/98 (65.3%) respondents indicated that they would test for HMPV. No HMPV-specific antiviral therapy was reported by 76/98 (77.6%) and no immunisation option by 64/95 (67.4%). Among 85 respondents to the management item, 33/85 (38.8%) selected IVIG for hypogammaglobulinaemia and 31/85 (36.5%) selected supportive therapy. Following a positive HMPV result, 26/96 (27.1%) would discontinue empiric antibiotics, 65/96 (67.7%) would continue them, and 5/96 (5.2%) were uncertain (Figure 2).

### 3.4. Case 2—RSV

You see a 62-year-old male patient, with a history of AML, now 2 years post allogeneic HCT, for routine follow-up in your outpatient clinic. He complains of headache, exertional dyspnoea, and a persistent heavy cough. He is an active smoker.

**Figure 3:**
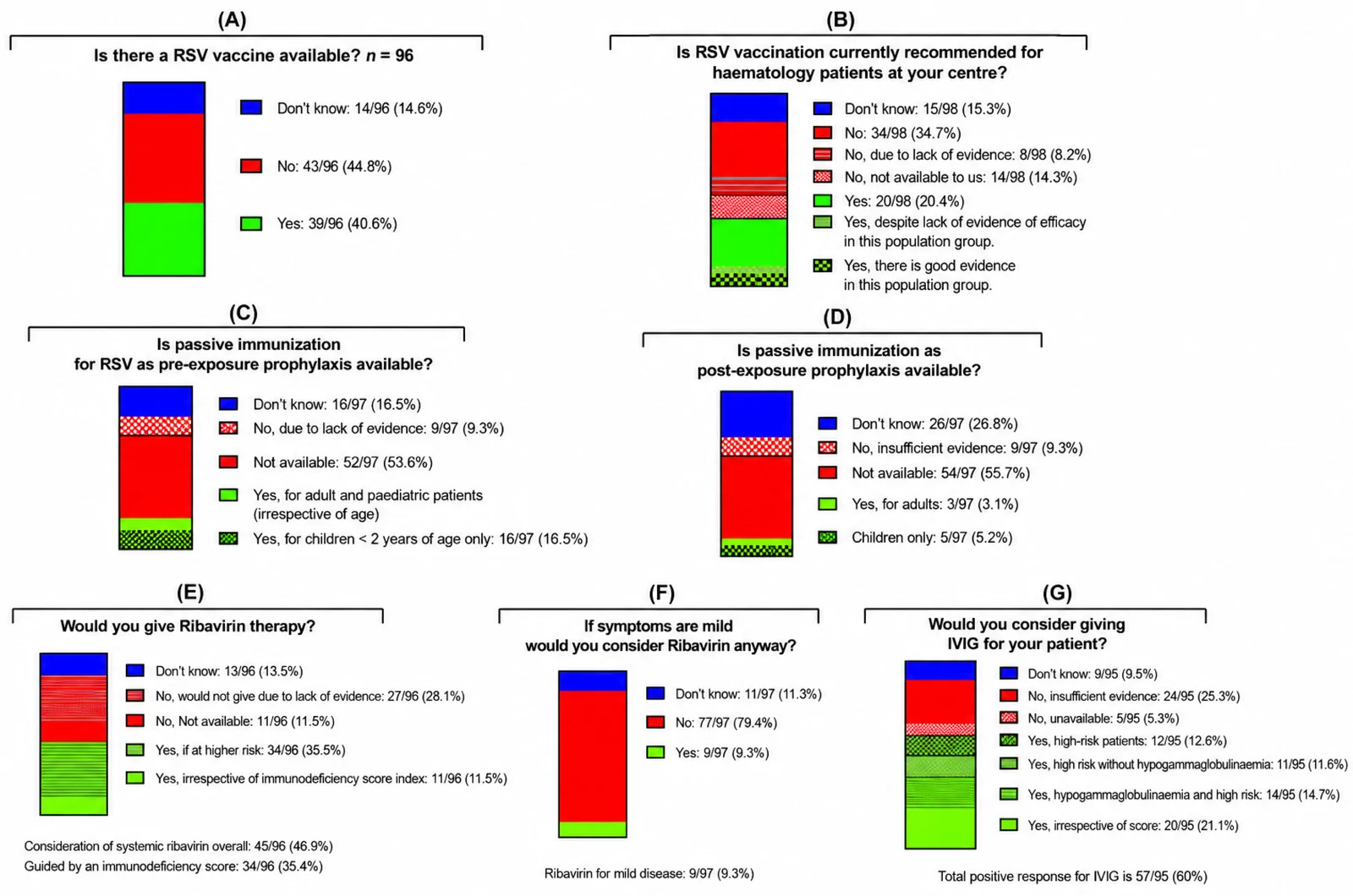
Respiratory syncytial virus (RSV) prevention and treatment practices. (**A**) Reported availability of an RSV vaccine; (**B**) reported recommendation of RSV vaccination for haematology patients at respondents’ centres; (**C**) reported availability of passive immunisation for RSV pre-exposure prophylaxis; (**D**) reported availability of passive immunisation for RSV post-exposure prophylaxis; (**E**) intended use of systemic ribavirin therapy; (**F**) intended use of ribavirin in patients with mild symptoms; (**G**) intended use of intravenous immunoglobulin (IVIG). Percentages use item-specific denominators.

RSV-related prevention and treatment practices varied among respondents. Active RSV vaccine availability was reported by 39/96 (40.6%), whereas adult passive prophylaxis was rarely reported (pre-exposure, 4/97 [4.1%]; post-exposure, 3/97 [3.1%]). Systemic ribavirin was considered by 45/96 (46.9%) respondents overall, including 34/96 (35.4%) when an immunodeficiency score indicated a high risk of progression; 9/97 (9.3%) would consider ribavirin for mild disease. IVIG was considered by 57/95 (60.0%), with responses reflecting hypogammaglobulinaemia, perceived risk of progression, perceived evidence and local availability (Figure 3).

### 3.5. Case 3—HPIV

You see a 79-year-old male with multiple myeloma on lenalidomide, daratumumab, dexamethasone therapy (two pathological fractures) presenting with a 10-day history of increasing fatigue, muscle aches, shortness of breath, no cough, but febrile (38.7 °C) in the emergency department (COVID-19 test: negative). Chest X-ray: Possible pneumonia left basally. He tests positive for HPIV.

**Figure 4:**
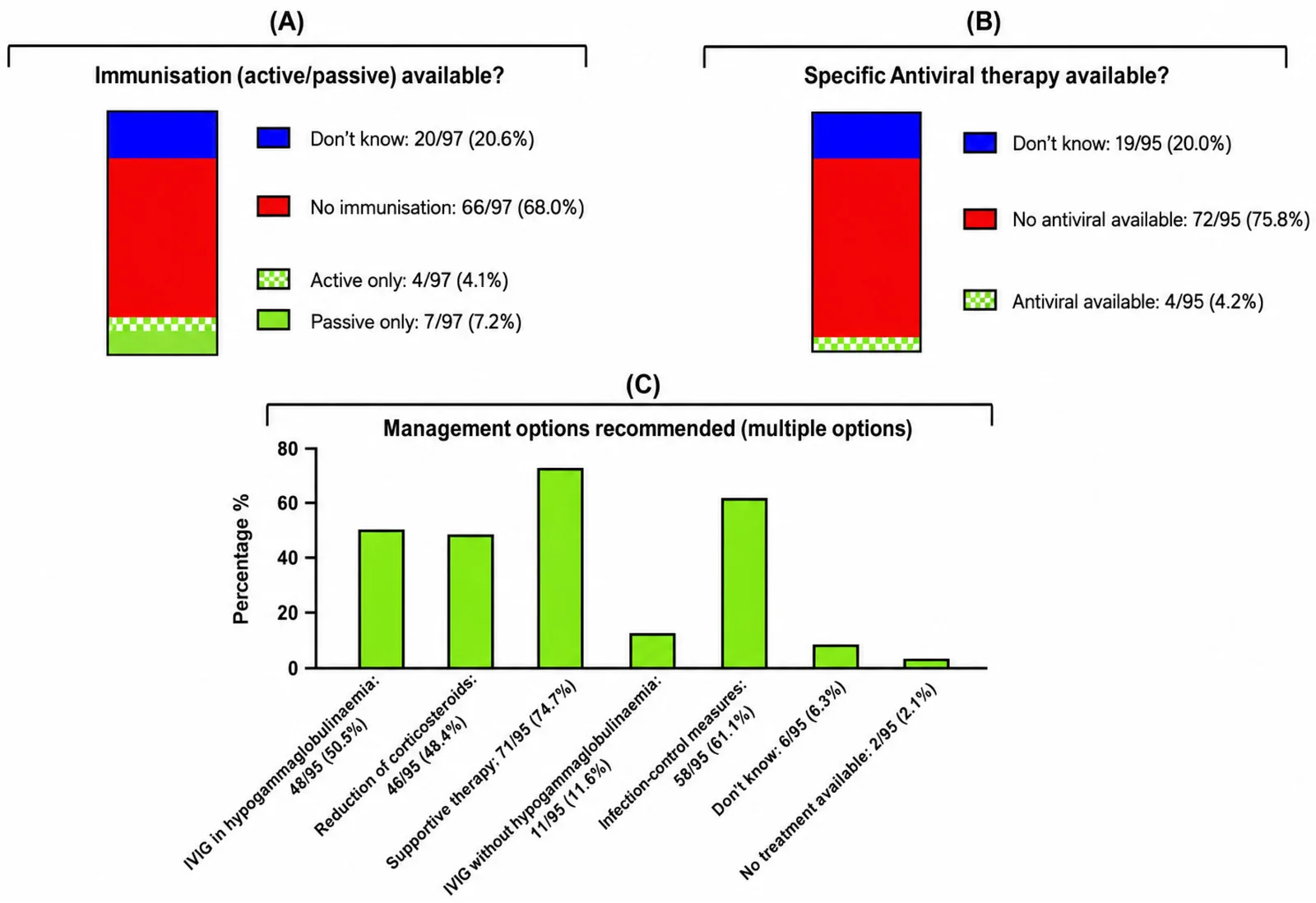
Human parainfluenza virus (HPIV) immunisation/antiviral availability and management. (**A**) Reported availability of active and/or passive immunisation against HPIV; (**B**) reported availability of HPIV-specific antiviral therapy; (**C**) intended management strategies after a positive HPIV result, including intravenous immunoglobulin (IVIG), reduction of corticosteroid therapy, supportive therapy, and infection-control measures. Panel C permitted multiple responses; percentages use item-specific denominators.

For the HPIV vignette, 66/97 (68.0%) respondents reported no available active or passive immunisation and 72/95 (75.8%) reported no specific antiviral therapy. Supportive therapy was selected by 71/95 (74.7%), infection-control measures by 58/95 (61.1%) and IVIG for hypogammaglobulinaemia by 48/95 (50.5%) (Figure 4).

### 3.6. Case 4—IAV

A 75-year-old female presented to the emergency department following an out-of-hospital cardiac arrest, presumed secondary to hypoxia after progressive respiratory deterioration. Her medical history included breast cancer (lumpectomy in 2008), peripheral arterial disease, chronic obstructive pulmonary disease (Global Initiative for Chronic Obstructive Lung Disease stage III), and newly diagnosed myelodysplastic syndrome. She tested positive for IAV.

**Figure 5:**
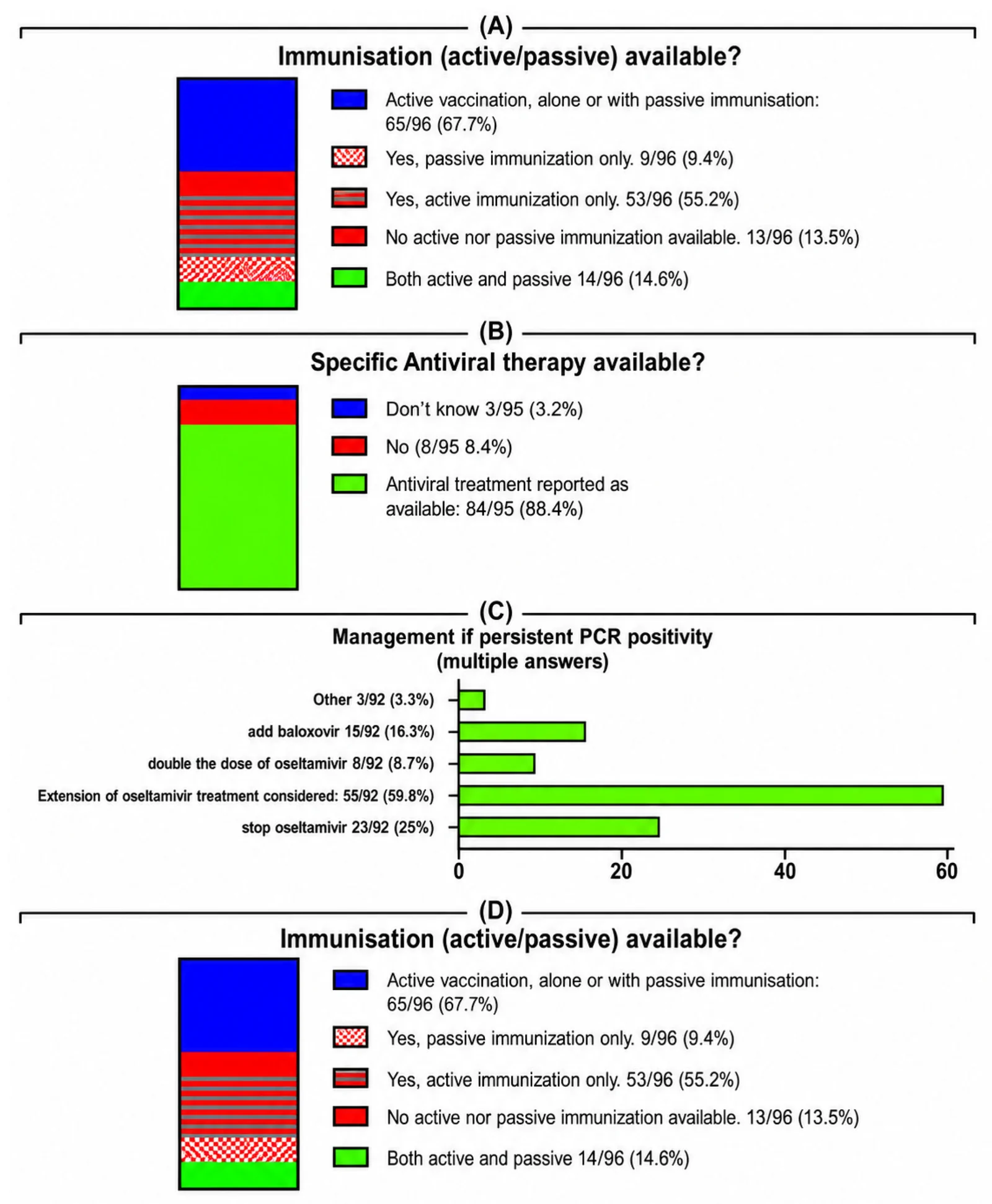
Influenza A virus (IAV) immunisation, antiviral access, post-exposure prophylaxis, and management of persistent polymerase chain reaction (PCR) positivity. (**A**) Reported availability of active and/or passive immunisation against IAV; (**B**) reported availability of IAV-specific antiviral therapy; (**C**) recommendation of oseltamivir for post-exposure prophylaxis; (**D**) intended management strategies for persistent IAV PCR positivity, including stopping oseltamivir, extending oseltamivir treatment, doubling the oseltamivir dose, adding baloxavir, or other approaches. (**Panel D**) permitted multiple responses; percentages use item-specific denominators.

Active IAV vaccination, either alone or together with passive immunisation, was reported as available by 65/96 (67.7%) respondents. Specific antiviral therapy was reported as available by 84/95 (88.4%), and 68/96 (70.8%) respondents recommended oseltamivir for post-exposure prophylaxis. For persistent PCR positivity, 55/92 (59.8%) would extend oseltamivir treatment to 10 days; other reported approaches included discontinuing therapy, adding baloxavir or doubling the oseltamivir dose (Figure 5).

### 3.7. Comparison of Key Findings Across All Four Viral Scenarios

Management differed across viral scenarios. HMPV and HPIV were characterised mainly by limited immunisation and antiviral options, with greater reliance on supportive care and infection-control measures. In contrast, IAV had the greatest reported availability of vaccination and antiviral therapy, while RSV management showed more variable use of vaccination, passive prophylaxis, and ribavirin (Table 1).

## 4. Discussion

Within this international convenience sample, reported testing and intended management practices for CARVs in haematology and HCT were heterogeneous. The most clinically relevant pattern was virus-specific actionability: IAV results generally mapped to established prevention and antiviral pathways, and RSV results prompted variable risk-adapted decisions, whereas HMPV and HPIV results mainly informed supportive care and infection control. The four-virus comparison suggests that the value of a diagnostic panel depends not only on pathogen detection but also on whether a positive result is linked to a defined management pathway.

That distinction was also evident in antimicrobial stewardship. For HMPV, only 26/96 (27.1%) respondents would discontinue empiric antibiotics after a positive result, whereas 65/96 (67.7%) would continue them. Thus, viral detection alone did not reliably translate into antibiotic de-escalation in this high-risk population, possibly because clinicians remain concerned about bacterial co-infection and clinical deterioration. Rapid multiplex PCR has been associated with faster diagnosis and, in some adult cohorts, reduced antibiotic exposure [8], but our findings suggest that these benefits require explicit stewardship pathways rather than testing alone.

Within the surveyed sample, 65/98 (66.3%) respondents reported a standardised respiratory viral testing strategy and 55/98 (56.1%) reported POCT availability at their institutions. These findings describe the participating clinicians and their reported local practice. Because respondents were unevenly distributed across specialties, regions and country income groups, we did not perform inferential comparisons and cannot attribute differences in diagnostic infrastructure to any specific clinical or geographical characteristic. Harmonised diagnostic principles that can be adapted to local laboratory and isolation capacity remain relevant for future implementation studies.

Only 4/95 (4.2%) respondents reported routine use of the MD Anderson ISI or Basel framework [10,16], whereas 34/96 (35.4%) indicated that they would consider systemic ribavirin if an immunodeficiency score indicated a high risk of progression in the RSV vignette. The vignette did not contain all variables needed to calculate either score; this item therefore assessed stated willingness to consider a scoring framework rather than respondents’ formal application of a score to the case. Given the limited-quality evidence for ribavirin and heterogeneity in practice [11,12], ECIL-10 supports risk-adapted management rather than indiscriminate treatment [13]. Prospective studies using consistently defined risk frameworks may improve comparability of treatment decisions.

For HMPV and HPIV, respondents largely recognised the absence of established pathogen-specific therapy and relied on supportive care, infection-control measures and immune optimisation, consistent with current guidance [13,14]. RSV occupied an intermediate position: only about 40% of respondents reported vaccine availability at their institutions, adult passive prophylaxis was rarely reported, and ribavirin and IVIG use varied. Adult RSV vaccines have shown efficacy in older adults [17,18], but evidence specific to haematological malignancy and HCT remains limited, a gap also emphasised by ECIL-10 [13]. This helps explain why expanding prevention options have not yet produced uniform practice.

IAV was the most standardised scenario: 84/95 (88.4%) respondents reported antiviral access and 68/96 (70.8%) recommended oseltamivir post-exposure prophylaxis. Active vaccination availability was reported by 65/96 (67.7%), yet persistent PCR positivity still generated divergent intended treatment choices. This pattern is compatible with guidance supporting seasonal inactivated vaccination and early antiviral therapy in immunocompromised patients [13,19,20], while highlighting uncertainty in prolonged infection and repeat-testing scenarios.

Interpretation should account for the non-probability convenience-sampling strategy, the 15.1% contact-based response proportion and the marked geographical and economic imbalance, which introduce potential selection and non-response bias. Respondents were concentrated in Europe and high- and upper-middle-income countries, with no representation from low-income countries, and participants may have had greater interest or expertise in respiratory viral infections than non-respondents. Institution type, transplant-centre volume, annual patient load and respondent-level transplant practice were not collected. Institution identifiers were also unavailable, so multiple respondents may have originated from the same institution. The small and unevenly distributed respondent groups precluded reliable between-group inference. In addition, several questionnaire items combined related but distinct concepts, including treatment availability, perceived evidence, clinician knowledge and institutional recommendations; the HMPV IVIG response option was ambiguously worded in the administered questionnaire. The questionnaire underwent multidisciplinary expert review but not formal psychometric validation or quantitative content-validity assessment. Finally, responses reflect stated management of clinical vignettes rather than observed care or patient outcomes. Accordingly, the findings describe practices reported by the participating clinicians and should not be generalised as prevalence estimates of international practice.

In conclusion, this exploratory survey identifies areas for further study: the availability and implementation of rapid diagnostics, and the linkage between test results and virus- and risk-specific management pathways. Among surveyed clinicians, IAV showed the most consistent reported treatment and prevention approaches, whereas RSV showed greater therapeutic and preventive heterogeneity. Testing for HMPV and HPIV was reported primarily to guide infection-control measures, clinical monitoring and antimicrobial stewardship. These findings reflect clinician-reported intended practices and should not be interpreted as estimates of international practice or observed clinical outcomes.

## Figures and Tables

**Table 1:** Comparison of the four viral scenarios.

Virus	Testing/Prevention	Antiviral Therapy	Key Management Responses
**HMPV**	Test for HMPV: 64/98 (65.3%)	No specific antiviral: 76/98 (77.6%)	IVIG if hypogammaglobulinaemic: 33/85 (38.8%)
	No immunisation: 64/95 (67.4%)		Supportive therapy: 31/85 (36.5%)
**RSV**	RSV-positive case	Systemic ribavirin: 45/96 (46.9%)	IVIG considered: 57/95 (60.0%)
	Reported active vaccine availability: 39/96 (40.6%)	Risk-score guided: 34/96 (35.4%)	Ribavirin for mild disease: 9/97 (9.3%)
	Adult passive prophylaxis rare: pre 4/97 (4.1%); post 3/97 (3.1%)		
**HPIV**	HPIV-positive case	No specific antiviral: 72/95 (75.8%)	Supportive therapy: 71/95 (74.7%)
	No immunisation: 66/97 (68.0%)		Infection control: 58/95 (61.1%)
			IVIG if hypogammaglobulinaemic: 48/95 (50.5%)
**Influenza A**	Influenza A-positive case	Reported specific antiviral availability: 84/95 (88.4%)	Oseltamivir post-exposure prophylaxis: 68/96 (70.8%)
	Reported vaccination availability: 65/96 (67.7%)		Persistent PCR positivity: extend oseltamivir 55/92 (59.8%)

**Note.** Data are n/N (%), using item-specific valid-response denominators. Multiple-response items may exceed 100%. Scenarios used analogous rather than identical survey items. Abbreviations: HMPV, human metapneumovirus; RSV, respiratory syncytial virus; HPIV, human parainfluenza virus; IVIG, intravenous immunoglobulin; PCR, polymerase chain reaction.

## Data Availability

The data presented in this study are available from the corresponding author upon reasonable request.

## Supplementary Materials

The following supporting information can be downloaded at https://www.mdpi.com/article/10.3390/diagnostics16172786/s1. File S1: Questionnaire; Table S1: Characteristics of survey respondents (N = 98); Table S2: Geographic distribution of survey respondents (N = 98); Table S3: Exploratory subgroup comparisons.

## Abbreviations

The following abbreviations are used in this manuscript:

CARVs	community-acquired respiratory viruses
HCT	haematopoietic cell transplantation
HMPV	human metapneumovirus
RSV	respiratory syncytial virus
HPIV	human parainfluenza virus
IAV	influenza A virus
PCR	polymerase chain reaction
POCT	point-of-care testing
EBMT	European Society for Blood and Marrow Transplantation
SASCETS	South African Stem Cell Transplant Society
ISI	Immunodeficiency Scoring Index
AML	acute myeloid leukaemia
IVIG	intravenous immunoglobulin
ECIL-10	10th European Conference on Infections in Leukaemia
