# Supplementary material for: Clinician-Reported Respiratory Viral Testing and Intended Management of Community-Acquired Respiratory Viral Infections in Haematology and Haematopoietic Cell Transplantation: An International Exploratory Survey
Source: Diagnostics (Basel). 2026 Aug 30;16(17):2786. doi: 10.3390/diagnostics16172786 (PMC13565004; doi:10.3390/diagnostics16172786)
Supplement: Supplementary file 1 [file diagnostics-16-02786-s001.zip › diagnostics-4386731-supplementary.pdf]

File S1: Questionnaire:

**Testing policies for Haematology patients: Respiratory Infections and Virus knowledge across multiple cEntres (THRIVE-study)**

Dear clinician,
We are doing research about the different testing policies of community acquired respiratory viruses (CARVs) specifically in haematology patients (with haematological malignancies receiving treatment and/or bone marrow transplant) across different centres in the world and the knowledge base of its management.
The purpose of this study is to better understand the heterogeneity in testing policies across the different centres and differences in the knowledge base among clinicians.
Your participation in this study can contribute to improve the testing policies and education of clinicians to improve management of vulnerable haematology patients that acquire CARVs.
The study will be conducted in the form of an online digital questionnaire. See link below.
Participation is entirely voluntary and no identifying information will be collected.
This study has been approved by the Human Research Ethics Committee of Spaarne Gasthuis, the Netherlands. The principal function of this Committee is to safeguard the rights and dignity of all human subjects who agree to participate in a research project and the integrity of the research.
If you have any concern over the way the study is being conducted, please contact the Supervisor Dr Marco Goeijenbier, who may be contacted on telephone number +31232240000, or by e-mail on m.goeijenbier@spaarnegasthuis.nl.
To obtain any additional information regarding this study please contact Dr Justin du Toit at justin@bonemarrowtransplant.co.za or +27799081872.
By filling out the questionnaire, you automatically consent to participating in this study.

**General Questions**

1. If haematology patients (with haematological malignancies receiving treatment and/or bone marrow transplant) who present themselves with respiratory complaints at the emergency department, does your hospital run a standard test strategy (i.e. are all patients tested for the same pathogens)
☐ Yes
☐ No
☐ Don't know

2. Is there the availability of point-of-care (POC) testing for these patients (test performed in the emergency department with results within 3 hours)?
☐ Yes
☐ No
☐ Don't know

(if Q2=yes)
3. Which pathogens are included in the POC test?
☐ RSV
☐ SARS-CoV-2
☐ Influenza
☐ other, namely
☐ Don’t know

(if Q2=yes)
4. if POC is negative, do you test further for other respiratory pathogens?
☐ Yes
☐ No
☐ Don’t know

(If Q4=yes)
5. What would you include in your respiratory panel if you decide to perform molecular diagnostics after the point-of-care test?
☐ SARS-CoV-2
☐ Influenza
☐ Respiratory Syncytial Virus (RSV)
☐ Human Metapneumovirus (hMPV)
☐ Parainfluenza viruses
☐ Bocavirus
☐ Rhinovirus
☐ Mycoplasma pneumoniae
☐ Adenovirus
☐ Legionella pneumophila
☐ Chlamydophila pneumoniae
☐ Bordetella pertussis
☐ Other, namely…
☐ All of the above
☐ Don’t know

6. For haematology patients with respiratory complaints (with haematological malignancies undergoing treatment and/or bone marrow transplant), do you perform an immunodeficiency score (Basel or MD Anderson scoring system) to judge progression of disease?
☐ Yes
☐ No
☐ Don’t know

We would now like to present you some real live cases to better understand the heterogeneity in testing policies across the different centre

Case 1 – Human Metapneumovirus (hMPV)
A 55-year-old female presents to the emergency department with progressive dyspnea. Her medical history includes hypertension, a history of Acute Myeloid Leukemia treated with an allogeneic stem cell transplantation, and chronic immunosuppression for graft-versus-host disease (GVHD).

This patient tests positive for Human Metapneumovirus (HMPV).
Questions
7. Based on your centres testing policy, would you have tested this patient for HMPV?
☐ Yes
☐ No
☐ Don’t know

8. Is specific antiviral therapy for prophylaxis or treatment available for HMPV in your medical centre?
☐ Yes
☐ No
☐ Don’t know

9. Is active or passive immunization for HMPV available currently?
☐ Yes
☐ No
☐ Don’t know

10. Which treatment or treatments are recommended by your centre for HMPV currently? Can choose more than one
☐ intravenous immunoglobulin with hypogammaglobulinaemia
☐ intravenous immunoglobulin without hypogammaglobulinaemia (< 4.5g/L) only
☐ judicious evaluation of lowering corticosteroid treatment
☐ administer ribavirin alone without IVIG
☐ administer ribavirin in combination with IVIG

11. Would you discontinue empiric antibiotic therapy based on the positive HMPV result?
☐ Yes
☐ No
☐ Don’t know

Case 2 RSV
You see a 62-year-old male patient, past history of AML, now 2 years post allogeneic stem cell transplant, for routine follow-up in your outpatient clinic. He mentions complaints of headache, exertional dyspnea, and a persistent heavy cough. He is an active smoker.

He tests positive of RSV.

12. Is there a vaccine available against RSV (active immunization) at your centre?
☐ Yes
☐ No
☐ Don’t know

13. Is RSV vaccination currently recommended for haematology patients at your centre?
☐ Yes, despite lack of evidence of efficacy in this population group.
☐ Yes, there is good evidence in this population group.
☐ No, due to lack of evidence.
☐ No, not available to us.
☐ Don’t know

14. Is passive immunization (monoclonal antibodies like Palivizumab) for RSV as pre-exposure prophylaxis available at your centre?
☐ Yes, for adult and paediatric patients (irrespective of age)
☐ Yes, for children < 2 years of age only
☐ No, due to lack of evidence
☐ Not available
☐ Don’t know

15. Is passive immunization (monoclonal antibodies like Palivizumab) for RSV as post-exposure prophylaxis available at your centre?
☐ Yes, for adult and paediatric patients (irrespective of age)
☐ Yes, for children < 2 years of age only
☐ No, due to lack of evidence
☐ Not available
☐ Don’t know

16. Would you consider giving systemic ribavirin therapy for the above patient?
☐ Yes, if immunodeficiency score index indicate that patient is at high risk of progression to lower respiratory tract infection.
☐ Yes, irrespective of immunodeficiency score index
☐ No, would not give due to lack of evidence
☐ Not available
☐ Don’t know

17. If symptoms are mild and patient is clinically well, would you consider systemic Ribavirin therapy anyway?
☐ Yes
☐ No
☐ Don't know

18. Would you consider giving IVIG for your patient? LRTI = lower respiratory tract infection.
☐ Yes, if immunodeficiency score index indicate that patient at high risk of progression to LRTI (or established LRTI) (not hypogammaglobulinaemic)
☐ Yes, if immunodeficiency score index indicate that patient at high risk of progression to lower respiratory tract infection (or established LRTI) (patient is hypogammaglobulinaemic)

☐ Yes, irrespective of immunodeficiency score index
☐ No, would not give due to lack of evidence
☐ Not available
☐ Don't know

Case 3 Parainfluenza

You see a 79-year-old male with myeloma on Lenalidomide, Daratumumab, Dexamethasone therapy (two pathological fractures) presenting with 10 day history of increasing fatigue, muscle aches, shortness of breath, no cough, but febrile (38.7 °C) in emergency department (COVID-19 test: negative). Chest X-ray: Possible pneumonia left basally. He tests positive for human parainfluenza (HPIV) virus.

19. Is there active and/or passive immunisation available against HPIV ?
☐ Yes, active immunization only.
☐ yes, passive immunization only.
☐ yes, both active and passive immunization only.
☐ No active nor passive immunization available.
☐ Don't know

20. Is there a specific antiviral therapy available against HPIV?
☐ Yes
☐ No
☐ Don't know

21. Management options for this patient includes: can choose more than one.
☐ IVIG if patient hypogammaglobulinaemic.
☐ Judicious lowering of corticosteroid treatment.
☐ Supportive therapy
☐ IVIG even if patient is not hypogammaglobulinaemic
☐ No treatment available

☐ Infection control measures as adopted by your centre (good personal hygiene, private rooms, personal protection measures in hospital (gloves, gowning and masks) for health-care providers and visitors.
☐ Don't know
Other, namely…

Case 4 Influenza A

You see a 75-year-old female in the emergency department with left-sided breast cancer (2008) - lumpectomy, known with peripheral arterial disease, COPD GOLD III and newly diagnosed Myelodysplastic syndrome (MDS) with Out-of-hospital cardiac arrest, most likely due to hypoxia but suspected respiratory deterioration leading up to event.

She tests positive for Influenza A virus (IAV).

22. Is there active and/or passive immunisation available against IAV ?
☐ Yes, active immunization only.
☐ yes, passive immunization only.
☐ Both active nor passive immunization available.
☐ No active nor passive immunization available.
☐ Don't know

23. Is there a specific antiviral therapy available against IAV?
☐ Yes, oseltamivir therapy is available
☐ No
☐ Don't know

24. Is post exposure prophylaxis with Oseltamivir (within 48 hours) recommended for haematology patients?
☐ Yes, recommended
☐ No, not recommended.
☐ Don't know

25. If above patient continues to test positive for IAV on nucleic acid testing (NAT) testing after 7 days with continuing symptoms, would you consider the following: choose more than one.
☐ Stop Oseltamivir and just supportive treatment.
☐ Extend oseltamivir for at least 10 days of therapy.
☐ Double the dose of Oseltamivir to 150mg twice daily.
☐ Consider adding Baloxavir therapy to Oseltamivir.
Other, namely..

Demographic Information
Age: ___________________________
Medical specialty: ________________

- Intensive care specialists or trainee
- Emergency medicine specialist or trainee
- Medical oncology or trainee
- Clinical haematology or trainee
- Paediatric oncologist or trainee
- Paediatric haematology or trainee

Years of experience or study: ______________
Country of residence: _________________________

Thank you for your participation and watch out for a link soon illustrating the research results.

**Table S1. Characteristics of survey respondents (N = 98)**

| Characteristic | Category | n | % of N |
|---|---|---|---|
| Specialty | Clinical haematology or trainee | 64 | 65.3% |
| | Medical oncology or trainee | 8 | 8.2% |
| | Intensive care specialist or trainee | 7 | 7.1% |
| | Emergency medicine specialist or trainee | 4 | 4.1% |
| | Paediatric oncology or trainee | 4 | 4.1% |
| | Paediatric haematology or trainee | 2 | 2.0% |
| | Missing/not reported | 9 | 9.2% |
| World region | Europe | 63 | 64.3% |
| | Africa | 19 | 19.4% |
| | South America | 6 | 6.1% |
| | North America | 4 | 4.1% |
| | Asia | 3 | 3.1% |

| Characteristic | Category | n | % of N |
|---|---|---|---|
| | Oceania | 2 | 2.0% |
| | Country missing | 1 | 1.0% |
| World Bank income group | High income | 69 | 70.4% |
| | Upper-middle income | 27 | 27.6% |
| | Lower-middle income | 1 | 1.0% |
| | Low income | 0 | 0.0% |
| | Country missing/unclassifiable | 1 | 1.0% |
| Clinical experience | Median (IQR), years | 15 (8.75–25) | 96 responses |
| Country | 26 identifiable countries | See Supplementary Table S1 | 97 country responses |

*Footnotes: Country names were standardized before assigning world region and World Bank FY2026 income category. Percentages use N=98 unless otherwise stated.*

**Table S2. Geographic distribution of survey respondents (N = 98)**

| World region | Participating countries (n) | Respondents, n | Respondents, % |
|---|---|---|---|
| Europe | Belgium (6); Croatia (1); Czech Republic (1); Denmark (1); France (6); Germany (5); Italy (17); Lithuania (1); Netherlands (16); Portugal (1); Serbia (1); Slovakia (1); Spain (2); Sweden (1); Switzerland (1); United Kingdom (2) | 63 | 64.3 |
| Africa | Algeria (1); Ghana (1); South Africa (17) | 19 | 19.4 |
| South America | Argentina (2); Brazil (3); Chile (1) | 6 | 6.1 |
| North America | Canada (1); United States (3) | 4 | 4.1 |
| Asia | Türkiye (3) | 3 | 3.1 |
| Oceania | Australia (2) | 2 | 2.0 |
| Not reported | Country not reported | 1 | 1.0 |
| **Total** | | **98** | **100.0** |

*Note: Percentages use all 98 analytic respondents as the denominator. One respondent (1.0%) did not report country. Country names were standardized before classification. Türkiye was classified in Asia (Western Asia).*

**Supplementary Table S3. Exploratory subgroup comparisons**

**S3A. By clinical specialty**

| Group | n | Standardized test | POCT | Immunodeficiency score | Test HMPV | Any ribavirin | Oseltamivir PEP |
|---|---|---|---|---|---|---|---|
| Clinical haematology | 64 | 42/64 (65.6%) | 34/64 (53.1%) | 3/64 (4.7%) | 40/64 (62.5%) | 29/62 (46.8%) | 39/63 (61.9%) |

| Group | n | Standardized test | POCT | Immunodeficiency score | Test HMPV | Any ribavirin | Oseltamivir PEP |
|---|---|---|---|---|---|---|---|
| Medical oncology | 8 | 6/8 (75.0%) | 5/8 (62.5%) | 0/8 (0.0%) | 7/7 (100.0%) | 6/8 (75.0%) | 7/8 (87.5%) |
| Intensive care | 7 | 4/7 (57.1%) | 5/7 (71.4%) | 0/6 (0.0%) | 5/7 (71.4%) | 1/7 (14.3%) | 6/7 (85.7%) |
| Emergency medicine | 4 | 3/4 (75.0%) | 3/4 (75.0%) | 0/4 (0.0%) | 1/4 (25.0%) | 0/4 (0.0%) | 3/4 (75.0%) |
| Paediatric oncology | 4 | 3/4 (75.0%) | 3/4 (75.0%) | 0/3 (0.0%) | 3/4 (75.0%) | 1/4 (25.0%) | 4/4 (100.0%) |
| Paediatric haematology | 2 | 0/2 (0.0%) | 0/2 (0.0%) | 0/2 (0.0%) | 0/2 (0.0%) | 1/2 (50.0%) | 2/2 (100.0%) |
| **Overall exact p** | | 0.5522 | 0.5455 | 1.0000 | 0.0444 | 0.0677 | 0.3724 |
| **Holm-adjusted p** | | 1.0000 | 1.0000 | 1.0000 | 0.2663 | 0.3383 | 1.0000 |

**S3B. By World Bank income group**

| Group | n | Standardized test | POCT | Immunodeficiency score | Test HMPV | Any ribavirin | Oseltamivir PEP |
|---|---|---|---|---|---|---|---|
| High income | 69 | 52/69 (75.4%) | 48/69 (69.6%) | 4/68 (5.9%) | 49/68 (72.1%) | 36/69 (52.2%) | 50/68 (73.5%) |
| Upper-middle income | 27 | 12/27 (44.4%) | 6/27 (22.2%) | 0/26 (0.0%) | 14/27 (51.9%) | 8/25 (32.0%) | 18/27 (66.7%) |
| Lower-middle income | 1 | 0/1 (0.0%) | 0/1 (0.0%) | 0/1 (0.0%) | 0/1 (0.0%) | 0/1 (0.0%) | 0/1 (0.0%) |
| **Overall exact p** | | 0.0048 | <0.0001 | 0.5893 | 0.0532 | 0.1050 | 0.2798 |
| **Holm-adjusted p** | | 0.0239 | 0.0003 | 0.5893 | 0.2130 | 0.3151 | 0.5597 |

**S3C. By world region**

| Group | n | Standardized test | POCT | Immunodeficiency score | Test HMPV | Any ribavirin | Oseltamivir PEP |
|---|---|---|---|---|---|---|---|
| Europe | 63 | 47/63 (74.6%) | 43/63 (68.3%) | 3/62 (4.8%) | 43/62 (69.4%) | 33/63 (52.4%) | 44/62 (71.0%) |
| Africa | 19 | 6/19 (31.6%) | 5/19 (26.3%) | 0/18 (0.0%) | 7/19 (36.8%) | 3/17 (17.6%) | 9/19 (47.4%) |
| Asia | 3 | 2/3 (66.7%) | 0/3 (0.0%) | 0/3 (0.0%) | 2/3 (66.7%) | 1/3 (33.3%) | 3/3 (100.0%) |
| North America | 4 | 3/4 (75.0%) | 3/4 (75.0%) | 1/4 (25.0%) | 4/4 (100.0%) | 3/4 (75.0%) | 4/4 (100.0%) |
| South America | 6 | 4/6 (66.7%) | 2/6 (33.3%) | 0/6 (0.0%) | 5/6 (83.3%) | 4/6 (66.7%) | 6/6 (100.0%) |
| Oceania | 2 | 2/2 (100.0%) | 1/2 (50.0%) | 0/2 (0.0%) | 2/2 (100.0%) | 0/2 (0.0%) | 2/2 (100.0%) |
| **Overall exact p** | | 0.0117 | 0.0015 | 0.3844 | 0.0399 | 0.0306 | 0.0636 |
| **Holm-adjusted p** | | 0.0586 | 0.0091 | 0.3844 | 0.1225 | 0.1225 | 0.1272 |

*Definition note: "Any ribavirin" includes any affirmative response to Q16 (risk-score guided or irrespective of risk score). "Affirmative" outcomes are compared against all other non-missing response categories. No transplant-versus-non-transplant clinician analysis is possible because transplant-practice status was not collected.*
